# Impact of body mass index on mortality rates in tuberculosis: A systematic review and meta-analysis

**DOI:** 10.12669/pjms.41.6.11722

**Published:** 2025-06

**Authors:** Yichen Li, Jie Cheng, Xinxin Bai, Jie Zheng, Peifang Mao, Xiuzhi Zhou

**Affiliations:** 1 Yichen Li Department of ICU, Wenzhou Central Hospital, Nanbaek Elephant Hospital, Wenzhou, Zhejiang Province 325000, P.R. China; 2 Jie Cheng Department of ICU, Wenzhou Central Hospital, Nanbaek Elephant Hospital, Wenzhou, Zhejiang Province 325000, P.R. China; 3 Xinxin Bai Department of ICU, Wenzhou Central Hospital, Nanbaek Elephant Hospital, Wenzhou, Zhejiang Province 325000, P.R. China; 4 Jie Zheng Department of ICU, Wenzhou Central Hospital, Nanbaek Elephant Hospital, Wenzhou, Zhejiang Province 325000, P.R. China; 5 Peifang Mao Department of ICU, Wenzhou Central Hospital, Nanbaek Elephant Hospital, Wenzhou, Zhejiang Province 325000, P.R. China; 6 Xiuzhi Zhou Department of ICU, Wenzhou Central Hospital, Nanbaek Elephant Hospital, Wenzhou, Zhejiang Province 325000, P.R. China

**Keywords:** Malnutrition, Obesity, Tuberculosis, Mortality

## Abstract

**Objective:**

To decipher the role of body mass index (BMI) measured at treatment initiation on mortality rates in tuberculosis (TB).

**Methods:**

This PROSPERO-registered PRISMA compliant review searched PubMed, Embase, CENTRAL, and Web of Science for studies from inception upto 10^th^ June 2024. Studies on adult patients examining mortality rates in TB patients based on BMI at treatment initiation were included. We analyzed crude and adjusted mortality rates in a random-effects meta-analysis model. Data was pooled to generate odds ratio (OR) and 95% confidence intervals (CI).

**Results:**

Ten studies were included. Both crude (OR: 2.54 95% CI: 2.13, 3.03 I^2^=56%) and adjusted (OR: 1.99 95% CI: 1.63, 2.44 I^2^=68%) data analysis showed that low BMI (<18.5 kg/m^2^) at treatment initiation was a significant factor increasing mortality rates of TB. Meta-analysis of crude data (OR: 1.07 95% CI: 0.68, 1.69 I^2^=76%) did not demonstrate a significant association between high BMI and mortality, but adjusted data showed that high BMI was associated with significantly reduced risk of mortality in TB patients (OR: 0.79 95% CI: 0.66, 0.95 I^2^=10%).

**Conclusions:**

Low BMI is associated with a significantly increased risk of mortality in TB patients. Scarce evidence also suggests that a high BMI may offer better survival rates for TB.

## INTRODUCTION

Tuberculosis (TB) is a major health challenge, with 10.4 million incident and 1.8 million deaths reported globally in 2015.^1^ Despite the commendable success of the Directly Observed Therapy, Short program in achieving an 85% treatment rate and reducing TB incidence globally,^2^ mortality rates among TB patients continue to be a significant concern.^3^,^4^ Numerous studies have focused on the effect of body mass index (BMI) on TB outcomes.^5^–^7^ BMI reflects the nutritional status of individuals, which, if unbalanced, may lead to compromised immune function that potentially impacts the efficiency of TB treatment.^8^ Low BMI, often indicative of undernutrition, may exacerbate the challenges of TB management through various mechanisms.

Moreover, patients with low BMI may experience delayed wound healing, increased susceptibility to infections, and a diminished capacity to withstand physiological stresses associated with TB and its treatment. These factors collectively contribute to a higher vulnerability to adverse postoperative outcomes, prolonged hospital stays, and increased mortality rates of TB patients.^6^,^7^,^9^ Therefore, understanding the potential link between BMI and TB outcomes has the potential to inform targeted interventions, refine treatment strategies, and enhance the overall efficacy of TB management protocols.

There is still no consensus on the link between different BMI categories and mortality risks in TB patients. This study aimed to summarize the existing evidence and explore how both low and high BMI may influence mortality rates in patients with TB.

## METHODS

This review and meta-analysis is in accordance with the PRISMA^10^ reporting guidelines. We registered the review protocol on PROSPERO (CRD42023494176) before beginning the review.

### Inclusion and exclusion criteria:

Studies selected for inclusion were those which were conducted on adult patients with TB. Studies were to stratify patients based on BMI (measured at treatment initiation) into any of the following groups: underweight, normal, and overweight or obese. Studies were to report mortality data based on these BMI groups. Studies reporting both crude and adjusted data were eligible. There was no restriction on study design, both prospective and retrospective cohort studies, case-control studies, and secondary analysis of randomized controlled trials were eligible. We also included patient-level meta-analysis studies provided that their data did not overlap with any other included studies. Studies were excluded if they were published only as abstracts, were from the same institute with overlapping or duplicate data, not stratifying the population based on BMI, and not reporting mortality data. In case of duplicate presentation of data, the study with a maximum sample size was included.

### Search strategy:

We explored the online repositories of PubMed, Embase, CENTRAL, and Web of Science for studies from inception up to 10^th^ June 2024. The search was restricted to English-language publications. We used the following search query was constructed using Boolean operators OR and AND to look for relevant articles:” (obese) OR (obesity[MeSH]) OR (overweight[MeSH])) OR (underweight)) OR (body mass index[MeSH])) OR (BMI) AND ((death[MeSH]) OR (mortality[MeSH]) AND (tuberculosis[MeSH])”. Two reviewers (JC and XB) searched independently and then combined the search results into a single reference manager software. Search results were screened by the software for duplicates which were excluded. The reviewers then read the titles and abstracts of the remaining studies to look for relevant articles. Selected studies underwent full-text analysis before final inclusion. The reviewers resolved any disagreements by consensus. We also screened the bibliography of included studies for any other missed studies.

### Data management:

A pre-defined data collection Form was used to collect the following data: study author, year of publication, country, study design, type of TB, sample size, patient characteristics (age, gender, human immunodeficiency virus (HIV) positive, treatment, BMI groups, their definition, mortality per group, adjusted covariates, and follow-up. We extracted both crude and adjusted mortality data for the meta-analysis.

### Study quality:

Two reviewers assessed the quality of studies using the tailored form of the Newcastle-Ottawa Quality Assessment Scale (NOS).^11^ Every study was assessed for selection bias, comparability of the exposed and unexposed groups, outcome assessment, and completeness of follow-up. The final score of each study can range from 0 meaning the highest risk of bias up to 9 meaning the lowest risk of bias.

### Statistical analysis:

Meta-analysis was done in “Review Manager” (RevMan, version 5.3; Nordic Cochrane Centre (Cochrane Collaboration), Copenhagen, Denmark; 2014). Mortality was compared between low BMI (underweight) vs normal BMI groups and high BMI (overweight/obese) vs normal BMI groups. Crude mortality rates were combined to generate odds ratio (OR) and 95% confidence intervals (CI). We also combined adjusted ORs obtained directly from the studies by log-transforming them in the generic inverse variance function of the software. A random-effects model was used for all analyses. The chi-square test and the I^2^ statistic indicated the heterogeneity between studies; I^2^>50% indicated substantial heterogeneity. Funnel plots were used to examine publication bias. Sensitivity analysis was conducted in the software itself to assess for any outliers. Certainty of evidence was examined using GRADE approach.

## RESULTS

### Search results:

**Fig.1:**
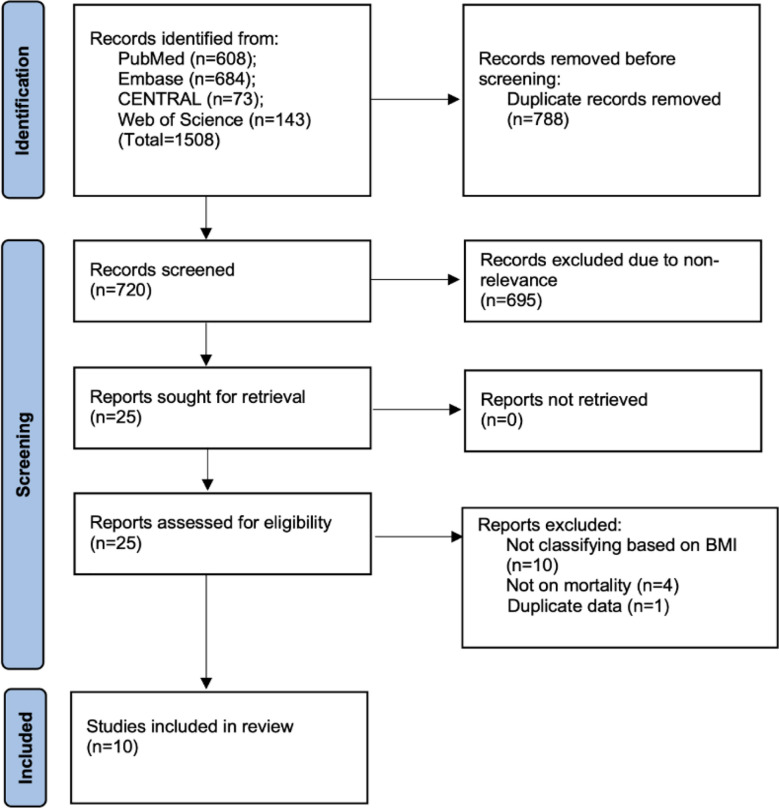
PRISMA flowchart depicting number of studies at each stage of the selection process.

We retrieved 1508 articles after the literature search. Finally, ten studies^5^-^7^,^9^,^12^-^17^ were found eligible for this meta-analysis Fig.1.

### Study characteristics:

The included studies were from the countries of Malawi, Taiwan, India, China, Korea, and Ethiopia. Table-I These were published between 2002 to 2024. Two studies were prospective studies while all others retrospective studies. One study was a patient-level meta-analysis that derived data from 52 studies which included patients treated between 1993 and 2016 in 37 countries/regions. This study was carefully chosen based on clear evidence that none of the other included studies were overlapping with this meta-analysis. Overall, the cumulative sample size of our review was 27,314. Two studies had a high proportion of HIV patients (80-100%). In the remaining studies, the proportion was <10%. Four studies classified patients only as underweight and normal BMI while the remaining studies also included overweight patients. Underweight or low BMI was uniformly defined as <18.5 kg/m^2^. Overweight was defined as ≥25 kg/m^2^ in all studies except one which defined it as ≥24.5 kg/m^2^. Seven studies presented adjusted data on mortality but with adjustment of variable confounders. Three studies were given a score of six on NOS, two studies received a score of seven, three got a score of eight and two were given a score of nine (Supplementary Table-I).

**Table-I:** Details of included studies. The authors may redesign Table by combining information in first three columns. It is too big cannot be accommodated even horizontally.

Study, location & Design	Type of TB	Sample size	Age (years)	Male (%)	HIV + (%)	Treatment	Groups	BMI (kg/m^2^)	Number	Mortality (%)	Adjusted covariates	Follow-up
Zachariah 2002^5^ Malawi R	Pul & EP	1181	37	49	80	2 months of supervised HRZS and 6 months of unsupervised HE	Underweight	<18.5	508	6.3	Gender, type of TB, HIV status, co-trimoxazole administration	4 weeks
							Normal	≥18.5	673	9.3		
Yen 2016^6^ Taiwan R	Pul & EP	1608	64.6	67.5	0.7	NR	Underweight	<18.5	405	24.4	Age, sex, education, unemployment, ESRD, malignancy, smear positivity, pleural effusion	Up to treatment completion
							Normal	18.5-24.9	1019	14.2		
							Overweight	≥25	184	10.3		
Yen 2017^7^ Taiwan R	Pul & EP	2410	64.5	67.1	NR	NR	Underweight	<18.5	607	24.2	BMI, age, sex, marital status, education level, unemployment, alcohol use, diabetes mellitus, ESRD, malignancy, TB relapse, AFB smear, TB culture, cavities on chest X-ray, EP TB and directly observed treatment	Up to treatment completion
							Normal	18.5-24.9	1525	14		
							Overweight	≥25	278	10.4		
Kornfeld 2020^9^ India R	Pul	389	NR	80	0	Based on Revised National Tuberculosis Control Program standards	Underweight	<18.5	184	6.5	NR	Up to treatment completion
							Normal	≥18.5	205	1		
Campbell 2022^12^ Multi-centic IPD meta-analysis	Pul	5148	37	66.1	8.8	NR	Underweight	<18.5	1702	12.9	Age, sex, year of treatment initiation, extent of disease, previous treatment, diabetes mellitus, country-level income, resistance to fluoroquinolones, resistance to second-line injectables	Up to treatment completion
							Normal	≥18.5	3446	4.4		
Xu 2022^13^ China R	Pul	1847	56	68.8	0.4	NR	Underweight	<18.5	640	1.7	NR	During hospitalization
							Normal	≥18.5	1207	0.5		
Bhargava 2023^14^ India P	Pul	2800	40.3	70.7	0.3	NR	Underweight	<18.5	2291	3.9	NR	6 months
							Normal	18.5-24.9	459	1.5		
							Overweight	≥25	29			
Li 2023^15^ China R	Pul	1809	47.6	NR	NR	2 months of HRZE followed by four months of HR	Underweight	<18.5	444	9.4	Sex, smoking, drinking, hypertension, hepatitis B, comorbidity, BMI, age, albumin, cholinesterase, C-reactive protein, lymphocyte count, and hemoglobin	3 years
							Normal	18.5-24.9	1196	3.4		
							Overweight	≥25	169	3.5		
Min 2023^16^ Korea P	Pul	9721	61.8	63.5	NR	NR	Underweight	<18.5	1927	19.3	Age, sex, current smoking status, heavy alcohol intake, and the presence of underlying comorbidities	Up to treatment completion
							Normal	18.5-22.9	4965	10		
							Overweight	≥23	2829	8.2		
Kegne 2024^17^ Ethiopia R	Pul & EP	401	35	56.6	100	NR	Underweight	<18.5	131	19.3	Asthma, diabetes, WHO stage, opportunistic infection, functional status, treatment adherence, marital status, co-trimoxazole preventive therapy, site of TB	212 days
							Normal	18.5-24.5	191	7.3		
							Overweight	>24.5	49	28.6		

P, prospective; R, retrospective; HIV, human immunodeficiency virus; BMI; H, isoniazid; R, rifampicin; Z, pyrazinamide, S, streptomycin; E, ethambutol; IPD, individual patient data; TB, tuberculosis; Pul, pulmonary; EP, extrapulmonary; NR, not reported; Newcastle Ottawa scale; ESRD, end-stage renal disease; AFB, acid-fast bacilli; WHO, World Health Organization.

**Supplementary Table-I:** Risk of bias analysis

Study, Location & Design	Selection of cohort	Comparability of groups	Outcomes assessment	NOS score
Zachariah 2002^5^, Malawi R	4	2	1	7
Yen 2016^6^, Taiwan R	4	2	2	8
Yen 2017^7^, Taiwan R	4	2	2	8
Kornfeld 2020^9^, India R	4	-	2	6
Campbell 2022^12^, Multi-centic IPD meta-analysis	4	2	2	8
Xu 2022^13^, China R	4	-	2	6
Bhargava 2023^14^, India P	4	-	2	6
Li 2023^15^, China R	4	2	3	9
Min 2023^16^, Korea P	4	2	3	9
Kegne 2024^17^, Ethiopia R	4	2	1	7

NOS, Newcastle Ottawa Scale

### Mortality rates of TB with low vs normal BMI:

**Fig.2:**
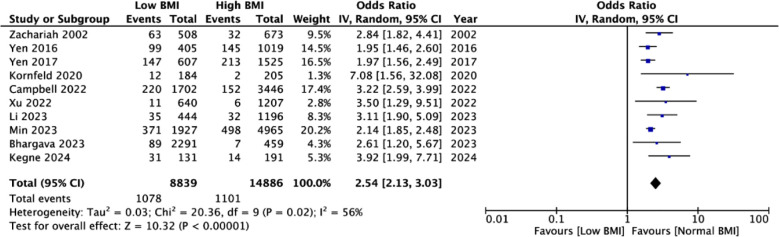
Meta-analysis of crude mortality rates between low vs normal BMI TB patients.

**Supplementary Fig.1:**
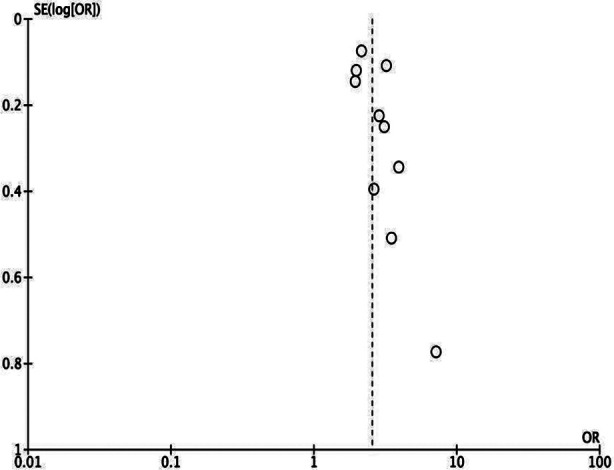
Funnel plot for meta-analysis of crude mortality rates between low vs normal BMI TB patients.

**Fig.3:**
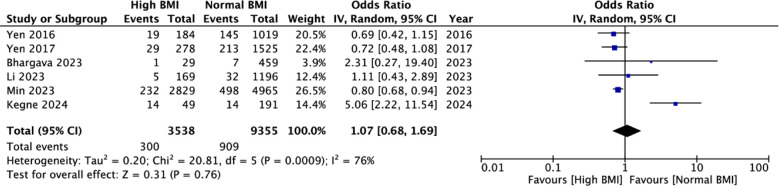
Meta-analysis of adjusted mortality rates between low vs normal BMI TB patients.

Meta-analysis showed a statistically significant increased risk of mortality in the underweight group (OR: 2.54 95% CI: 2.13, 3.03 I^2^=56%) indicating that underweight patients had higher mortality rates as compared to normal BMI patients. Fig.2 No publication bias was noted. Supplementary Fig.1 Leave-one-out analysis did not alter the significance of results on the exclusion of any study. Seven studies reported adjusted data. Pooled analysis showed a statistically significant association between low BMI and risk of mortality in TB (OR: 1.99 95% CI: 1.63, 2.44 I^2^=68%) indicating that underweight patients had higher mortality rates as compared to normal BMI patients. Fig.3 Sensitivity analysis showed that the results were still significant on sequential exclusion of all studies. Certainty of evidence based on GRADE was found to be low (Supplementary Table-II).

**Supplementary Table-II:** GRADE assessment of evidence

	Crude mortality Low vs normal BMI	Adjusted mortality Low vs normal BMI	Crude mortality High vs normal BMI	Adjusted mortality High vs normal BMI
Number of studies	10	7	6	4
***Downgrade quality of evidence***				
Risk of bias	Serious*	Serious^	Serious*	Serious^
Inconsistency	No	No	No	No
Indirectness	No	No	No	No
Imprecision	No	No	No	No
***Publication bias***				
Upgrade quality of evidence				
Large effect	No	No	No	No
Plausible confounding	No	No	No	No
Dose-response	No	No	No	No
Overall certainty of Evidence	Low	Low	Low	Low

* NOS score ranged from 6-9 in the included studies,^NOS score ranged from 7-9 in the included studies

### Mortality rates of TB with high vs normal BMI:

**Fig.4:**
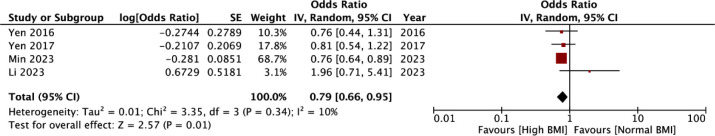
Meta-analysis of crude mortality rates between high vs normal BMI TB patients.

**Supplementary Fig.2:**
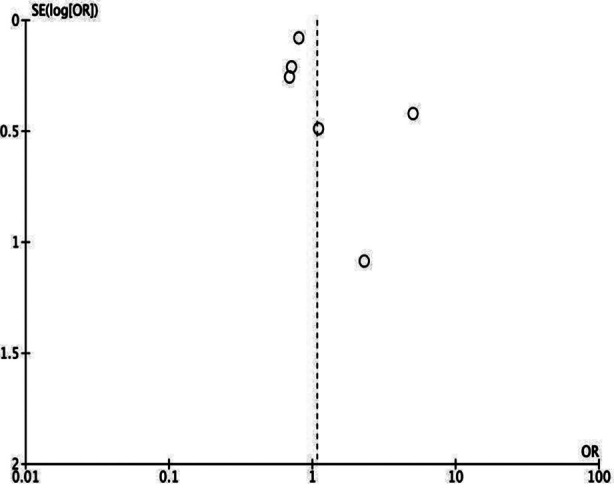
Funnel plot for meta-analysis of crude mortality rates between high vs normal BMI TB patients.

**Fig.5:**
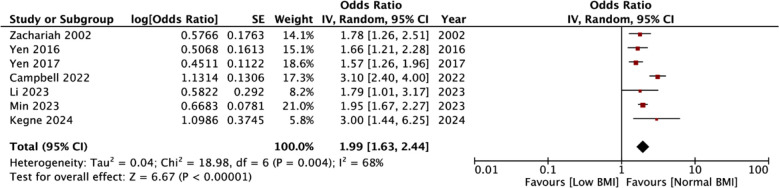
Meta-analysis of adjusted mortality rates between high vs normal BMI TB patients.

No significant association was noted between high BMI and mortality in TB (OR: 1.07 95% CI: 0.68, 1.69 I^2^=76%) indicating that there was no difference in mortality rates of obese patients as compared to normal BMI patients. Fig.4 No publication bias was noted. Supplementary Fig.2 On the exclusion of Kegne et al,^17^ the results showed significantly better survival in the high BMI group (OR: 0.79 95% CI: 0.69, 0.91 I^2^=0%). Only four studies reported adjusted data. Meta-analysis showed that high BMI was associated with a significantly reduced risk of mortality as compared to normal BMI in TB patients (OR: 0.79 95% CI: 0.66, 0.95 I^2^=10%) indicating that obese patients had lower mortality rates as compared to normal BMI patients. Fig.5 These results were unstable on sensitivity analysis and turned non-significant on the sequential exclusion of all studies except Li et al.^15^ Certainty of evidence based on GRADE was found to be low (Supplementary Table-II).

## DISCUSSION

TB remains a persistent global health challenge that requires continuous research to better understand various factors, influencing treatment outcomes and mortality rates.^18^ The results of our meta-analysis of 10 studies showed that lower BMI correlated with significantly higher mortality risk in TB patients. Analysis of studies reporting data on overweight patients also revealed a significant decrease in mortality risk in patients with higher BMI.

It is pertinent to note that two types of data were analyzed in our meta-analysis. Crude data analysis showed that low BMI was associated with a 2.5 times increased risk of mortality in TB while no significant association was noted between high BMI and mortality. However, an outlier study^17^ was noted in the later analysis, the exclusion of which showed that high BMI was linked with a 21% reduced risk of mortality. The meta-analysis of crude data should be interpreted with caution as there are several confounders that can influence tuberculosis mortality.

Research has shown that older age, male gender, unemployment, low income, substance abuse, mental disorders, lung diseases, HIV positivity, recurrent TB, and delayed visits are all risk factors for increased mortality in TB.^19^,^20^ While not all of these factors were reported and adjusted by the included studies, an attempt was made to pool more reliable data in the form of adjusted ORs. Meta-analysis of the same showed that low BMI was still associated with a two-fold increased risk of mortality while high BMI was associated with a 21% reduction in the risk of death.

Previous studies have showed that low BMI, indicative of undernutrition, is a potential risk factor for adverse TB outcomes since malnutrition can contribute to delayed wound healing, increased susceptibility to infections, etc.^12^ Therefore, this increased vulnerability of underweight patients may result in adverse postoperative outcomes, prolonged hospital stays, and ultimately increased mortality rates among TB patients.^21^–^23^ In contrast, patients with higher BMI, reflecting better nutritional status, may benefit from a more robust immune response, quicker recovery, and increased resilience to the challenges posed by TB and the treatment.^6^,^7^ This protective effect may reduce the risk of mortality in individuals with higher BMI, highlighting the intricate interplay between nutritional status and the body’s ability to combat TB and its complications.^24^

Notably, we noted marked heterogeneity in the meta-analysis of low BMI, which may be explained by variations in sample size, population demographics, comorbidities, and duration of follow-up. One important factor is the variation in the HIV positivity rates among the included studies. HIV and TB have been known to influence each other’s natural history and pathogenesis, thereby increasing the risk of HIV-TB co-infection.^25^ HIV is the strongest known risk factor for active TB and is the most common opportunistic disease among HIV-infected patients. It is also one of the most important factors increasing the risk of TB mortality.^26^ While the HIV positivity rate varied in the studies, the forest plot showed a consistent trend of significant association between low BMI and mortality in all included studies. This was noted for both crude and adjusted mortality data, indicating that low BMI is an independent risk factor for TB mortality.

The recognition of BMI as a potential determinant of TB mortality has significant implications for clinical practice. Identifying patients with low BMI during TB diagnosis may prompt clinicians to implement tailored nutritional interventions alongside standard treatment protocols. Interventions that specifically aim at malnutrition may enhance immune function, and improve overall patient resilience to the challenges posed by TB and its treatment.^27^ While our meta-analysis provides valuable insights, further research is essential to validate the clinical utility of BMI as a predictor of survivability in patients with TB. Prospective studies with standardized data reporting are needed to strengthen the robustness of evidence and guide clinicians in adopting effective strategies for improved outcomes in TB cases. Additionally, exploring the impact of BMI on TB treatment response and the development of complications warrants further investigation to comprehensively address the complex interplay between nutritional status and TB outcomes.

While our study primarily focused on BMI, we acknowledge that malnutrition encompasses a broader spectrum of indicators.^28^,^29^ In-depth exploration of other markers of malnutrition, such as serum albumin levels, micronutrient deficiencies, or anthropometric measurements beyond BMI, could further enhance our understanding of the multifaceted relationship between nutritional status and TB outcomes. Future investigations should consider incorporating these additional markers to provide a comprehensive evaluation of the nutritional landscape in TB patients.

### Limitations:

Variations in sample size introduce a degree of heterogeneity and the potential for biases within our analysis. Furthermore, the retrospective cohort design of all included studies may limit our ability to definitively establish causation, due to the inherent risk of confounding variables. Difference in follow-up within studies is another potential limitation, as it can introduce bias and affect the generalizability of findings. The timing of assessment of outcome was dependent on the follow-up time and it is plausible that studies with short follow-up may have missed the outcome. Some of the included studies also included a mix of pulmonary and extra-pulmonary TB which increased heterogeneity. Disseminated form of TB is associated with higher mortality and given the lack of data, we could not separate outcomes of pulmonary and extra-pulmonary TB. Lastly, several factors like sex, HIV status, comorbidities, smoking, alcohol use, drug resistance, and bacterial load can influence the risk of mortality. Due to lack of data from the individual studies, we were unable to segregate data based on these important variables.

The link between low BMI and increased risk of mortality underscores the integral role of addressing nutritional status in TB management. Clinicians should actively monitor BMI of all patients with TB to optimize patient outcomes. Appropriate nutritional management strategies may help normalize BMI and improve patient outcomes.

## CONCLUSION

Our meta-analysis shows a low BMI may be associated with a significantly increased risk of mortality in TB patients. Scarce evidence also suggests that a high BMI may lead to better survival in TB patients. HIV positivity can be an important confounder which can influence mortality rates and needs further investigations. Further prospective studies with standardized data reporting are needed to enhance the evidence base and to allow clinicians to adopt more informed and effective strategies to improve outcomes of this patient population.

### Authors’ Contributions:

**YL:** Study design, literature search and manuscript writing.

**JC, XB, JZ, PM and XZ:** Data collection, data analysis, literature search, critical review.

YL was involved in the manuscript revision and validation and is responsible for the integrity of the study.

All authors have read and approved the final manuscript.
